# Response to “Do not miss Bickerstaff encephalitis as a complication of SARS-CoV-2 vaccines”^☆^

**DOI:** 10.1016/j.radcr.2022.05.057

**Published:** 2022-06-22

**Authors:** Fatemeh Yarmahmoodi, Pooya Iranpoor

**Affiliations:** a Medical Imaging Research Center, Shiraz University of Medical Sciences, Shiraz, Iran; b Department of Radiology, Shiraz University of Medical Sciences, Shiraz, Iran

*Dear editor*,

In response to the letter entitled “Do not miss Bickerstaff encephalitis as a complication of SARS-CoV-2 vaccines” [1], we would like to thank Finsterer et al. for their attentiveness to our study [2].  In the letter to the editor by Finsterer et al. [1], some concerns have been expressed about the misdiagnosis of acute, disseminated encephalomyelitis (ADEM) in the case reported in our paper [2].

First, they declared that nerve conduction studies (NCSs) should have been performed on the patient to determine if there was a proximal demyelinating or axonal lesion. In the primary assessment of the patient's CSF, 2 white blood cells and 56 mg of protein were observed, which ruled out the central nervous system infections (meningitis) and led the diagnosis toward Guillain-Barre Syndrome (GBS); however, in the follow-ups of the patient's symptoms and other evaluations using MRI, the diagnosis of ADEM was made, and MRI findings such as nonenhanced nerve roots and presence of supratentorial lesions led us to this diagnosis. Therefore, for the patient with an ADEM diagnosis, NCS was not performed.

**Fig. 1:**
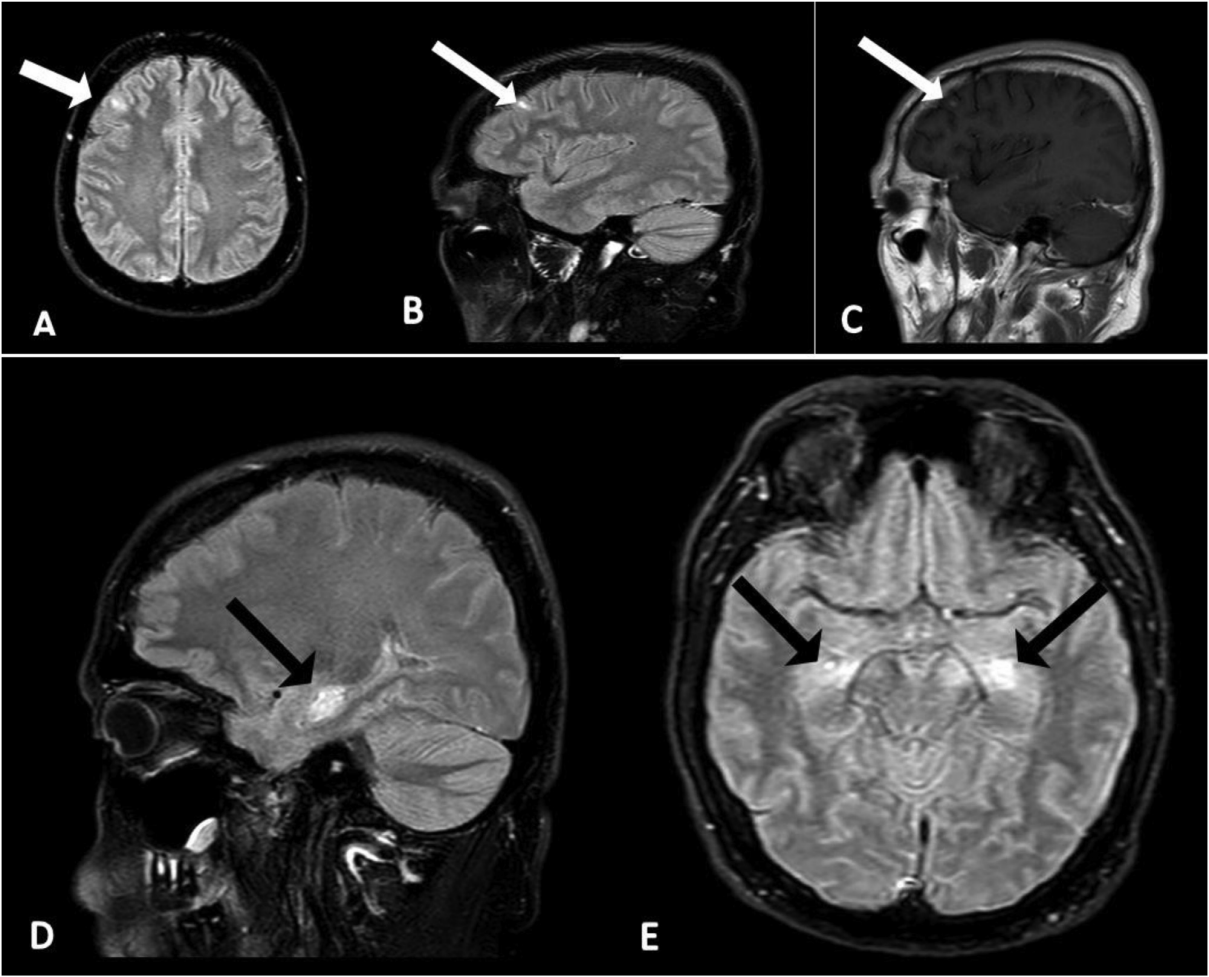
(A,B) Axial and sagittal FLAIR images demonstrate high signal focus in subcortical part of RT frontal lobe. (C) Sagittal postcontrast T1 image shows enhancement )white arrow). (D,E) Sagittal and axial FLAIR images have increase signal of bilateral mesial temporal lobes (black arrows).

Second, It was mentioned that our MRI findings do not comply with ADEM since ADEM diagnosis should accompany supra- and infra-tentorial lesions. Our case report did not state that supratentorial lesions were not seen. Actually, supratentorial lesions were observed in the right frontal lobe and bilateral mesial temporal lobes (more dominated on the left side) (Fig. 1).

Third, they pointed out that brainstem Bickerstaff encephalitis (BBE) has been informed as a complication of SARS-CoV-2 vaccines. We should reply that we have complete information regarding BBE as a complication of SARS-CoV-2 vaccines [3], [4], [5], and we considered it during our survey and making the diagnosis.

Fourth, it has been mentioned [1] that unilateral lesions do not justify facial diplegia and quadriparesis. As declared in the previous part, bilateral lesions were observed in MRI images; however, the number of lesions on the left side was more, and no enhancement was seen in nerve roots in post-contrast images. These findings explain facial diplegia and quadriparesis.

Fifth, some questions were discussed regarding the tendon reflexes. In our case, tendon reflexes had been preserved, and no hyper or hypo changes occurred during the illness.

Sixth, there were some questions about the time of appearance and disappearance of the lethargy and myalgia. In response, we should say that according to the patient's report, lethargy and myalgia started gently 2 weeks after vaccination. The patient might have reported his primary muscle weakness as lethargy and myalgia. However, the severity of lethargy and myalgia gradually increased, and after a month, it was accompanied by other symptoms such as quadriparesis and continued until the onset of quadriparesis despite supportive treatments.

Seventh, there was a question about the time of disappearance of corticospinal tract lesions. With the administration of corticosteroids, all the patient's symptoms had improved, so the patient was discharged, and in the follow-up MRI, which was done as OPD, all the lesions had improved.

Finally, the authors thank the editor for providing this opportunity for us to go into more detail about our case report.
